# Dietary Patterns Associated with Cognitive Function among the Older People in Underdeveloped Regions: Finding from the NCDFaC Study

**DOI:** 10.3390/nu10040464

**Published:** 2018-04-09

**Authors:** Zhaoxue Yin, Jing Chen, Jian Zhang, Zeping Ren, Kui Dong, Virginia B. Kraus, Zhuoqun Wang, Mei Zhang, Yi Zhai, Pengkun Song, Yanfang Zhao, Shaojie Pang, Shengquan Mi, Wenhua Zhao

**Affiliations:** 1 National Institute for Nutrition and Health, Chinese Center for Disease Control and Prevention, 27 Nanwei Road, Xicheng District, Beijing 100050, China; yinzx@chinacdc.cn (Z.Y.); zhangjian@ninh.chinacdc.cn (J.Z.); spk_8210@163.com (P.S.); shaojiepang@126.com (S.P.); 2 Division of Non-Communicable Diseases Control and Community Health, Chinese Center for Disease Control and Prevention, 155 Changbai Road, Changping District, Beijing 102206, China; zhaiiahz@163.com; 3 Shanxi Center for Disease Control and Prevention, 8 Xiaonanguan Street, Taiyuan 030012, China; 13753122807@163.com (J.C.); zpr0504@163.com (Z.R.); 4 Linyi Center for Disease Control and Prevention, 1159 Shuangtanan Road, Linyi 044100, China; lyjkzxmbk@163.com; 5 Duke Molecular Physiology Institute and Division of Rheumatology, Department of Medicine, Duke University School of Medicine, 300 North Duke St, Durham, NC 27701, USA; kraus004@duke.edu; 6 National Center for Chronic and Non-Communicable Diseases Control and Prevention, Chinese Center for Disease Control and Prevention, 27 Nanwei Road, Xicheng District, Beijing 100050, China; wangzhuoqun1016@163.com (Z.W.); zhangmei@ncncd.chinacdc.cn (M.Z.); zhaoyanfang@ncncd.chinacdc.cn (Y.Z.); 7 College of Biochemical Engineering, Beijing Union University, 18 Zone three, Fatouxili, Chaoyang District, Beijing 100023, China; msq365@hotmail.com

**Keywords:** dietary pattern, cognitive function, factor analysis, older adults

## Abstract

Although dietary patterns are crucial to cognitive function, associations of dietary patterns with cognitive function have not yet been fully understood. This cross-sectional study explored dietary patterns associated with cognitive function among the older adults in underdeveloped regions, using 1504 community-dwelling older adults aged 60 and over. Diet was assessed using a food frequency questionnaire and 24-h dietary recall. Factor analysis was used to extract dietary patterns. Global cognitive function was assessed using the Mini-Mental State Examination (MMSE). Two dietary patterns, a “mushroom, vegetable, and fruits” (MVF) pattern and a “meat and soybean products” (MS) pattern, were identified. The MVF pattern, characterized by high consumption of mushrooms, vegetables, and fruits was significantly positively associated with cognitive function (*p* < 0.05), with an odds ratio of (95% CIs) 0.60 (0.38, 0.94) for cognitive impairment and β (95% CIs) 0.15 (0.02, 0.29) for –log (31-MMSE score). The MS pattern, characterized by high consumption of soybean products and meat, was also associated with better cognitive function, with an odds ratio of 0.47 (95% CIs 0.30, 0.74) for cognitive impairment and β (95% CIs) 0.34 (0.21, 0.47) for –log (31-MMSE score). Our results suggested that both the MVF and MS patterns were positively associated with better cognitive function among older adults in underdeveloped regions.

## 1. Introduction

About 47 million people live with dementia worldwide; the number is projected to rise to more than 131 million by 2050 [1]. Rates of dementia have increased steadily in the past two decades in China [2]. Dementia and cognitive impairment are the major causes of functional dependence and mortality [3] and hence, result in a huge economic and care burden on society, especially as there are no effective treatments or drugs to modify the course of dementia currently. Therefore, one of the most effective strategies for preventing dementia is believed to involve the exploration and management of modifiable risk factors for cognitive impairment, such as the dietary factors studied here. 

Many studies have investigated the association of nutrients and food groups, such as antioxidant vitamins and n-3 polyunsaturated fatty acids, with cognitive function; results have been inconsistent [4]. In contrast to the focus on a single nutrient or food group, diet should be considered as in the context of the ‘whole’ dietary pattern, consisting of a complex of various foods and nutrients, the combination of which may act synergistically to provide stronger health effects than their individual components. Dietary patterns have been the focus of recent studies evaluating the association of nutrition with health outcomes or diseases such as type 2 diabetes [5] and heart disease [6]. Dietary patterns are also believed to be one promising strategy to investigate the link between food and cognitive decline [7]. The methods of exploring dietary patterns include an a priori approach (for example, Healthy Diet Index), a posteriori approach (principal components analysis, factor analysis, and cluster analysis), and reduced rank regression, a new method used efficiently in nutrition epidemiology; this method can derive the dietary patterns associated with selected response variables that have known relations with a disease [8]. In contrast to the a priori method, the main advantage of the a posteriori methods is that they take into account complex correlations of the “food matrix” and they are not hypothesis-driven food groups choices, and so they are particularly valuable for exploring new and specific dietary recommendations among a population. Using the a priori approach [7], some studies reported cognitive protection in association with specific dietary patterns such as the Mediterranean diet [9] and the Dietary Approaches to Stop Hypertension (DASH) [10]. However, few studies have identified dietary patterns associated with cognition using an a posteriori approach.

Although several studies investigating associations of dietary patterns and cognitive function have been conducted in Western countries or other countries in Asia [11,12,13], to the best of our knowledge, studies focusing on dietary patterns and cognition among Chinese older adults are very scarce [14]. The accumulation of more evidence across various countries is very important for dementia prevention because beneficial dietary patterns identified in one population may not be relevant for another because of the great difference in cooking style, dietary habits, food items, and nutrients in foods. 

Furthermore, the associations of dietary patterns with cognition can be influenced by the socioeconomic characteristics of the study populations, such as income, the overall quantity of the diet, and the corresponding energy intake [11,15]. Although older people of lower socioeconomic status are most sensitive to both the detrimental and beneficial impacts of diet, few studies have focused on this subgroup.

To address this knowledge gap, we investigated associations of dietary patterns with cognitive function among the Chinese older adults in underdeveloped regions using the Nutrition and Chronic Disease Family Cohort (NCDFaC) study.

## 2. Materials and Methods

### 2.1. Study Participants

The NCDFaC study was established in the Shanxi province, based on the China National Nutrition and Health Survey (CNNHS), which was conducted in 2002 [16]. Participants of the CNNHS in six poor counties in the Shanxi province were invited to join in the NCDFaC as a follow-up in 2015 (at a time when they were still underdeveloped). Cross-sectional data from the 2015 follow-up survey, the first to document cognitive function, was used for this study. Of the 1645 older people who participated in the 2015 assessment, 98 participants were excluded due to missing data on cognitive function. In addition, 43 participants were further excluded due to missing values for important covariates, resulting in 1504 participants (aged 60–90 years) included in this study. There were no significant differences in mean cognitive function scores between those participants included in this study and the 43 excluded participants (*p* = 0.31).

The NCDFaC study was approved by the ethics committee of the National Institute for Nutrition and Health of the Chinese Center for Disease Control and Prevention, and written informed consents were obtained from all participants (or their proxies).

### 2.2. Assessment of Cognitive Function

Global cognitive function was assessed using the validated Chinese version of the Mini-Mental State Examination (MMSE) that has been used in previous studies [17,18]. Due to the significant association of education level with performance on the MMSE test, validated education-based cutoff scores were used for the MMSE test to define cognitive impairment: 19/20 for those without formal education, 22/23 for those with 1–6 years of formal education (primary school), and 26/27 for those with more than 6 years of education (middle school or higher) [17,18].

### 2.3. Assessment of Dietary Intake

A 40-item semi-quantitative food frequency questionnaire (FFQ) was used to assess dietary intake over the prior 12 months; this questionnaire represented a slightly expanded version of the validated FFQ that was used in CNNHS in 2002 [19]. Participants were asked to report the consumption frequency of each food item or food group, followed by a question on the amount of consumption (grams for solid or milliliter for liquid food items). Consumption frequency was ascertained with the following questions: (1) how many times per day; (2) how many times per week; (3) how many times per month; or (4) how many times per year. Consumption frequency was transformed to mean consumption frequency of a day for responses related to times per week, month, or year. The dietary intake amount of each food item/group was calculated by multiplying the mean frequency of a day and amount of consumption per time. The 40 food items were classified into 24 food groups for factor analysis (Table S1), taking into account their nutritional characteristics and the grouping scheme used in other studies. In addition to the FFQ, the total energy intake (Kcal/day) was calculated based on 24-h dietary recall that was conducted at the same time as FFQ, using the Chinese food composition table [20].

### 2.4. Dietary Patterns Assessment

Exploratory factor analysis was used to extract dietary patterns based on the predefined 24 food groups; factor loadings were based on the dietary intake. The factors were rotated with varimax rotation to simplify the interpretation. The number of retained factors was based on an eigenvalue >1.00, scree plot test, and factor interpretability. Two main factors were identified and interpreted as dietary patterns; each pattern was named after the food groups with the highest loading (absolute value of loading >0.4). Food groups with absolute factor loading coefficients of 0.25 and above were considered to be strongly associated within a pattern. We calculated the standardized score of each factor for every participant by summing the consumption of each food group that was weighted by its factor loading [21].

### 2.5. Covariates

Data were collected through face-to-face interviews. Information was collected related to socio-demographics (age, sex, education, and marital status), lifestyles (smoking, alcohol drinking, and physical activities), energy intake, height, weight, blood pressure, diabetes, stroke, and activities of daily life (ADL) disability. 

Marital status was dichotomized as married or non-married, with non-married including those divorced, widowed, or never married. Alcohol drinking was defined as “yes” if one or more drinks of alcohol were consumed in the last 12 months. Total metabolic equivalents (METs) per week were estimated according to the frequency and intensity of physical activities; lower physical activity was defined as “yes” if one’s physical activity level was lower than the mean METs per week for the cohort. Systolic blood pressure (SBP) and diastolic blood pressure (DBP) were measured three times on the right arm in the sitting position; the mean values were used. Hypertension was defined as “yes” if anyone of the following criteria were met: SBP ≥140 mmHg, DBP ≥90 mmHg, self-reported diagnosed hypertension, or regular use of anti-hypertension drugs. Diabetes was defined as “yes” based on fasting serum glucose ≥7.0 or a self-report of a diabetes diagnosis. Stroke was defined by the question, “Have you ever been diagnosed with a stroke by the hospital?”. ADL disabilities were assessed using the Katz Index of Independence in Activities of Daily Living [22]. Participants were asked if they experienced difficulty in performing the following six activities: bathing, dressing, toileting, transfers, continence, and eating. ADL disability was defined as “yes” if they had difficulty in performing any one or more of these ADL tasks. Higher triglyceride was defined as “yes” if fasting triglycerides were ≥2.26 mmol/L. Higher cholesterol was defined as “yes” if the fasting level of total cholesterol was ≥6.22 mmol/L [23].

### 2.6. Statistical Analysis

Participant characteristics by cognitive impairment status were compared by *t*-test for continuous variables and by chi-square tests for categorical variables. Two dietary patterns were derived using factor analysis with a principal component method. For each dietary pattern identified, participants were categorized into quartiles according to the standardized dietary pattern score. The characteristics by quartiles were expressed as means and percentages for continuous and categorical variables, respectively, and linear trend analysis was conducted by general linear model or the Cochran–Mantel–Haenszel test for continuous and categorical variables, respectively, to investigate associations of characteristics with factor scores of each dietary pattern. 

Logistic regression was used to analyze associations of dietary patterns with cognitive impairment, in which the lowest quartile was defined as the reference group. Odds ratios (ORs) and 95% confidence intervals (CIs) were calculated, and linear trends of ORs were assessed. General linear regression analyses were used to estimate the β coefficient and 95% CIs of the association of MMSE scores with dietary patterns scores; MMSE scores were transformed to –log (31-MMSE score) because of a skewed distribution of the original MMSE score [24]. An unadjusted model and two models adjusted for covariates were fit for each dietary pattern: model 1 was adjusted for age, sex, education level, and marital status; model 2 was adjusted additionally for current smoking, alcohol drinking, physical activity status, energy intake, obesity, high triglyceride, high cholesterol, diabetes, hypertension, stroke, and ADL disability.

All statistical analyses were performed with SAS, version 9.3 (SAS Institute Inc., Cary, NC, USA). *p* < 0.05 was considered statistically significant, and all *p* values were two-sided. 

## 3. Results

### 3.1. Characteristics of the Participants

Based on comparisons of the 1504 subjects included in this study and the 141 excluded subjects, the prevalence of obesity was slightly lower among the excluded 141 participants (*p* = 0.045); there were no statistically significant differences in age, sex, education, prevalence of lower physical activities, smoking, alcohol drinking, diabetes, hypertension, stroke, or ADL disability (*p* > 0.10). The characteristics of the included participants by cognitive status are listed in Table 1. Compared to those with normal cognition, those with cognitive impairment were more likely to be older, lack formal education, be physically inactive, have ADL disability, and less likely to be married. 

### 3.2. Identification of Dietary Patterns

We identified two major dietary patterns using factor analysis; the factor loading matrices for the two patterns are listed in Table 2. The first dietary pattern, “mushroom, vegetables, and fruits” (MVF), was characterized by high consumption of fresh mushrooms, dried mushrooms, vegetables, fruits, and legumes and moderate consumption of grains, nuts, and soybean milk. The second dietary pattern, “meat and soybean products” (MS), was characterized by high consumption of soybean products, livestock meat, poultry meat, organ meats, and aquatic products, and less intake of other plant-based foods. The two dietary patterns explained 56.5% and 38.4% of the variation in food intake, respectively.

### 3.3. Distribution of Characteristics by Dietary Patterns Scores Quartiles

As showed in Table 3, compared to participants with the lowest quartile score of the MVF pattern, those in the higher quartiles were more likely to be younger, married, less likely to be female and lack of formal education, less likely to report insufficient physical activity or ADL disability (*p*_trend_ < 0.01), and more likely to have higher energy intake and salt intake (*p*_trend_ < 0.01). Increasing prevalence of hypertension and diabetes were correlated with increasing MVF scores (*p*_trend_ < 0.01).

Compared to participants with the lowest quartile score of the MS pattern, those in the higher quartiles were more likely to be married, less likely to be female and illiterate (*p*_trend_ < 0.001), and more likely to have higher energy intake and lower salt intake (*p*_trend_ < 0.01). The prevalence of stroke, hypertension, and diabetes were negatively associated with the scores of the MS pattern by quartile (*p*_trend_ < 0.01) (Table 3). 

### 3.4. Associations of Dietary Patterns with Cognitive Function

Compared to participants in the lowest quartile of the MVF pattern, the risk of cognitive impairment of participants in the highest quartile was statistically significantly lower (*p* < 0.05); the adjusted (Model 2) OR was 0.60 (95% CIs 0.38, 0.94), with a statistically significant trend across quartiles (*p* = 0.03) (Table 4). 

Compared to participants in the lowest quartile of the MS pattern, the risk of cognitive impairment of participants in the highest quartile was statistically significantly lower (*p* < 0.001); the fully adjusted (Model 2) OR was 0.47 (95% CIs 0.30, 0.74), with *p* for trend of 0.001 (Table 4). 

**Figure 1:**
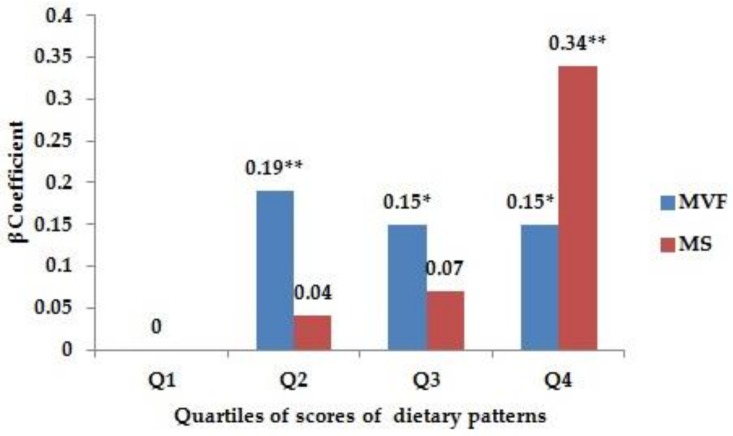
Association of quartile scores for dietary patterns with cognitive function using multiple linear regression ^a,b,c^. ^a^ data shown are β coefficients of quartile scores for cognitive function. ^b^ The original MMSE score was transformed as –log (31-MMSE score). ^c^ Models were adjusted for age, sex, education years, marital status, smoking, alcohol drinking, physical activity, energy intake, hypertension, diabetes, stroke, obesity, ADL disability, high cholesterol, and high triglyceride. * *p* < 0.05; ** *p* < 0.01; ADL: Activities of daily living; Q: Quartile; MVF = mushrooms, vegetables, fruits; MS = meat, soybeans; MMSE: Mini-Mental State Examination.

Based on multiple linear regression analyses, both dietary patterns were associated with cognitive performance, yielding β coefficients (95% CIs) from fully adjusted models (Model 2) of 0.15 (0.02, 0.29) and 0.34 (0.21, 0.47) for the highest quartile of the MVF and MS dietary patterns, respectively (Figure 1).

## 4. Discussion

In this study, the MVF and MS patterns were derived using factor analysis among Chinese older adults in underdeveloped regions; both of the patterns were significantly positively associated with better global cognitive function.

The MVF pattern identified in this study contains some of the same food components, such as vegetables, fruits, and legume, as those included in the most frequently studied Mediterranean Diet (MeDi), which has been shown to affect not only the risk of Alzheimer’s Disease but also of pre-dementia syndromes and their progression to overt dementia [9]. The MVF pattern was also somewhat consistent with a few other dietary patterns that have been associated with higher cognitive function including: a “health” dietary pattern characterized by high consumption of fruits, whole grains, fresh dairy products, vegetables, nuts, and fish [11]; a “[f]ruit and [v]egetable” pattern [13]; a “vegetable” pattern [14]; and a “[p]lant foods and fish” pattern [25]. 

These findings related to the MVF dietary pattern were consistent with the protective cognitive effect of fruit and vegetable intake reported in some prior studies [26], in which it was concluded that each increment of 100 g per day of fruit and vegetable consumption was related to an approximate 13% reduction in risk of cognitive impairment and dementia. The rich antioxidant micronutrient content and anti-inflammatory properties of fruits and vegetables may offer protection for cognition [27]. A cohort study [28] suggested that frequent mushroom consumption was significantly associated with cognitive function because of the natural, free radical scavengers and anti-oxidant and anti-inflammatory effects in mushrooms [29]. Higher total nut intake over the long term was associated with modestly better cognitive performance [30]; this might be due to the synergy and interaction of all of the nutrients and other bioactive components in nuts [31].

Interestingly, although the MVF pattern was associated with better cognition, it was also found to be associated with a higher prevalence of hypertension and diabetes. This association was likely linked to the increasing salt intake of the MVF quartiles and supported by data in this study showing that the mean salt intake increased from 7.2 g/day to 8.1 g/day across the participants in the MVF quartiles. Vegetables are traditionally boiled or stewed with salt in China, which is different from their preparation in Western countries [32]. Moreover, salt intake is one of the most important risk factors for hypertension and a risk factor for diabetes [33]. The link between vegetable consumption and higher salt intake has also been reported in Japan [34]. However, since diet can influence cognition through more than one pathway, the positive effect of the MVF dietary pattern for cognitive function may outweigh its negative effect.

Traditionally, red meat, a classical element of a Western diet, was thought to be associated with worse cognitive performance [35]. Staubo et al. found a negative association of red meat with inferior and superior parietal cortical thickness among older adults in the United States [36]. A diet represented by a high intake of red meat was shown to be independently associated with worse cognitive function in elder subjects aged 85 and over in the United Kingdom [37]. However, as some studies showed, this concept deserves a reassessment [38]. Some studies found no relationship between an “[a]nimal food” dietary pattern (high intake of meat, fish, and seasonings) and global cognitive function among Japanese elderly populations [25], and no association of a “meat-fish” pattern with the risk of cognitive impairment among older Hong Kong Chinese people [39]. The present study has suggested that the MS dietary pattern may also protect against cognitive impairment; this is consistent with one recent study that reported an independent association of a “meat” dietary pattern with protection against attention decline [14]. This beneficial effect of red meat on cognitive function could be related to some beneficial components of lean red meat (iron, protein, MUFA, and PUFA) [38]. Higher fish intake has also been associated with better cognitive function [38]; compared with individuals who consumed <1 serving of fish/week, the mean annual rate of global cognitive decline was reduced by 0.35 points (95% CIs 0.13, 0.58) among those consuming ≥1 serving/week [40]. Consistent with another study [25], soy products, important food components of the MS dietary pattern, were also found to be protective of cognition in our study.

The inconsistencies in associations of meat/animal food dietary patterns with cognitive function may arise from across study differences in total energy intake, as well as the tools used for assessing cognition and sample size. The participants in our study were from economically underdeveloped regions; they were characterized by a mean low daily energy intake of 1500 kcal/day (1602 kcal for males and 1392 kcal for females), which was much lower than those of their Japanese [25] and Hong Kong [39] counterparts in other studies. Notably, more than 80% of the subjects in our study failed to meet the energy intake recommendations of the Chinese Nutrition Association [41]. Thus, our results support the importance of energy intake for cognitive function. This is consistent with other studies showing that both the quality and the quantity of the diet impacted cognition [42] and that the beneficial effects of a healthy dietary pattern on cognitive performance were stronger in participants with lower energy intake [11]. In addition, the mean meat (including livestock meat and poultry meat) intake of the participants in the highest quartile of the MS dietary pattern is about 61 g/day, which is still in the recommended range (40–75 g/day) of the Chinese Nutrition Association [41], so the MS pattern derived in this study may ultimately be beneficial for health outcomes, including cognition function. Finally, the food items/groups across studies are usually not completely the same even if the dietary pattern derived in a different study had the same name, so the synergistic or antagonistic effect on cognition function was different, which may be another factor resulting in inconsistencies among various populations. 

Several limitations should be noted. First, our analyses were cross-sectional, therefore, we cannot infer a causal relationship. Second, although the 24-h dietary recall assessments that we used may not be as accurate as 3-day food records, they are still an acceptable means of assessing energy intake. Third, we could not adjust for the apolipoprotein E (APOE) status, while APOE4 is associated with increased risk of cognitive impairment [43], so associations of dietary patterns with cognitive impairment may potentially be overestimated. Nor could we adjust for socioeconomic status; however, we adjusted for education, marital status, and energy intake, which may serve as important indicators of socioeconomic status. In our multivariate statistics analysis, it was very helpful to adjust for the effect of socioeconomic status. Moreover, the participants included in this study were from those subjects enrolled in the 2002 survey, so the loss to follow-up bias may exist. However, the follow-up rate of this group of participants (aged 60 year and over in 2015) was 85.9%, which was relatively high, so the bias from the loss to follow-up would be minimized. Finally, our conclusions draw from Chinese subjects and may not be generalizable to Western populations due to differences in dietary habits.

This paper represents one of very few studies that investigate the relationships between dietary patterns and cognitive function in a relatively large sample of community-dwelling older adults from underdeveloped regions, which made it possible to observe these associations in an economically disadvantaged situation. In addition, we controlled for a broad range of health-related factors as potential confounders.

## 5. Conclusions

In conclusion, both the MVF and MS patterns were positively associated with cognitive function among older adults in underdeveloped regions. Our finding is very meaningful to dementia prevention for economically disadvantaged older adults, especially those with low energy intake. Further longitudinal studies are warranted to confirm this association.

## Figures and Tables

**Table 1:** Characteristics of study participants by cognitive status (*n* = 1504) ^a^.

Characteristics	Cognitive Impairment		*p* Value
	No	Yes	
No. of participants	1214	290	
Age (years), mean (SD)	67.8 (6.2)	72.9 (7.7)	<0.001
Female	563 (48.4)	152 (54.9)	0.10
Formal education level (years)			
0	99 (8.2)	55 (19.0)	<0.001
1–6	614 (50.6)	120 (41.4)	
>6	501 (41.3)	115 (39.7)	
Marital status	1034 (85.2)	223 (76.9)	<0.001
Current smoking	282 (23.2)	48 (16. 6)	0.01
Alcohol drinking	188 (15.5)	25 (8.6)	0.002
Lower physical activities	784 (64.6)	216 (74.5)	0.001
Energy intake	1544.2 (719.2)	1318.5 (686.4)	<0.001
Stroke	131(10.8)	38 (13.1)	0.26
Hypertension	746 (61.5)	173 (59.7)	0.57
Diabetes	147 (12. 1)	35 (12.1)	0.99
ADL disability	210 (17.4)	115 (35.0)	<0.001
Obesity	132 (10.9)	22 (7.6)	0.10
High triglyceride	181 (14.9)	37 (12.8)	0.35
High cholesterol	75 (6.2)	13 (4.5)	0.27

Abbreviations: ADL, activities of daily living; ^a^ Data are shown as *n* (%) for categorical variables, including female, formal education level, marital status, current smoking, alcohol drinking, lower physical activities, stroke, hypertension, diabetes, ADL disability, obesity, high triglyceride, and high cholesterol; and shown as mean (SD) for continuous variables, including age and energy intake.

**Table 2:** Factor loading for the two derived major dietary patterns.

Foods/Food Groups	MVF	MS
**Cereal and Grains**	0.375	−0.103
**Tubers**	0.298	0.062
**Fried foods**	−0.044	0.195
**Red meat**	0.136	0.558 ^a^
**Poultry meat**	0.048	0.572 ^a^
**Organ meat**	0.074	0.438 ^a^
**Aquatic products**	0.060	0.442 ^a^
**Milk**	0.230	−0.041
**Dairy products**	0.057	−0.061
**Eggs**	0.209	0.196
**Soybean products**	0.138	0.581 ^a^
**Soybean milk**	0.283	0.002
**Dried legumes**	0.406 ^a^	−0.066
**Vegetables**	0.493 ^a^	−0.181
**Pickles**	−0.008	0.061
**Fresh mushrooms**	0.496 ^a^	0.062
**Dried mushrooms**	0.483 ^a^	0.088
**Dessert**	−0.047	0.226
**Fruits**	0.443 ^a^	0.080
**Nuts**	0.329	0.093
**Alcoholic beverages**	0.21	0.070
**Fruit and vegetables juices**	0.199	0.130
**Beverage**	0.085	0.213
**Tea**	0.247	0.105
**% of explained variance**	56.5%	38.4%
**% of accumulated explained variance**	56.5%	94.9%

^a^ Absolute factor loadings >0.40; MVF = mushrooms, vegetables, fruits; MS = meat, soybeans.

**Table 3:** Characteristics of study participants by quartile categories of each dietary pattern score ^a^.

	MVF Dietary Pattern			MS Dietary Pattern		
	Q1	Q4	*p*_trend_	Q1	Q4	*p*_trend_
Age	70.6 (7.7)	66.6 (5.5)	<0.001	69.2 (6.6)	68.5 (7.0)	0.06
Female (%)	213 (56.7)	156 (41.5)	<0.001	208 (55.5)	159 (42.4)	<0.001
Lack of formal education	60 (16.0)	19 (5.1)	<0.001	48 (12.8)	23 (6.1)	0.10
Marital Status	305 (81.1)	344 (91.5)	<0.001	294 (78.4)	336 (89.4)	<0.001
Current smoking	79 (20.0)	80 (21.3)	0.82	67 (17.9)	107 (28.5)	<0.001
Alcohol drinking	43 (11.4)	81(21.5)	<0.001	46 (12.3)	72 (19.2)	0.006
Lower physical activities	282 (75.0)	205 (54.5)	<0.001	229 (61.1)	260 (69.2)	0.04
Energy intake	1299.7 (632.3)	1742.8 (760.1)	<0.001	1366.8 (601.4)	1753.7 (788.2)	<0.001
Salt intake	7.2 (2.60)	8.1 (3.2)	0.003	8.3 (3.5)	7.5 (2.8)	0.005
Stroke	37 (9.8)	48 (12.8)	0.29	67 (17.9)	25 (6.7)	<0.001
Hypertension	194 (51.6)	250 (66.5)	0.001	277 (73.9)	183 (48.7)	<0.001
Diabetes	39 (10.4)	56 (14.9)	0.02	62 (16.5)	31 (8.2)	<0.001
ADL disability	101 (27.2)	44 (11.8)	<0.001	80 (21.5)	61 (16.3)	0.06
Obesity	30 (7.98)	41 (10.90)	0.20	51 (13.6)	34 (9.0)	0.12
High TC	29(7.7)	19 (5.3)	0.14	19 (5.1)	19(5.1)	0.93
High TG	55 (14.6)	61 (16.2)	0.43	59(15.7)	48 (12.8)	0.38

^a^ Data are shown as *n* (%) for categorical variables, including female, lack of formal education, marital status, current smoking, alcohol drinking, lower physical activities, stroke, hypertension, diabetes, ADL disability, obesity, high triglyceride, and high cholesterol; and shown as mean (SD) for continuous variables, including age and energy intake. ADL: Activities of daily living; TC: total cholesterol; TG: Triglyceride; MVF = mushrooms, vegetables, fruits; MS = meat, soybeans; Q: Quartile.

**Table 4:** Associations of dietary patterns scores with cognitive impairment using logistic regression ^a,b^.

	Quartiles of Dietary Patterns				*p*_trend_
	Q1	Q2	Q3	Q4	
*MVF dietary pattern*					
Unadjusted	1.00	0.68 (0.48, 0.96) *	0.56 (0.39, 0.80) **	0.38 (0.26, 0.57) **	<0.001
Model 1	1.00	0.68 (0.47,1.01)	0.68 (0.46,1.01)	0.53 (0.34, 0.81) **	0.004
Model 2	1.00	0.70 (0.48, 1.02)	0.70 (0.47, 1.04)	0.60 (0.38, 0.94) *	0.03
*MS dietary pattern*					
Unadjusted	1.00	0.78 (0.55, 1.11)	0.63 (0.44, 0.90) *	0.44 (0.30, 0.65) **	<0.001
Model 1	1.00	0.76 (0.52, 1.10)	0.70 (0.48, 1.03)	0.47 (0.31, 0.71) **	<0.001
Model 2	1.00	0.70 (0.48, 1.03)	0.68 (0.46, 1.01)	0.47 (0.30, 0.74) **	0.001

^a^ Data are shown as ORs (95% CI) of quartile groups for cognitive impairment. ^b^ Q1 was the reference group. * *p* < 0.05; ** *p* < 0.01; Model 1: adjusted for age, sex, education years, and marital status; Model 2: additionally adjusted for smoking, alcohol drinking, physical activity, energy intake, hypertension, diabetes, stroke, obesity, ADL disability, higher cholesterol, and higher triglyceride. Q: Quartile; MVF = mushrooms, vegetables, fruits; MS = meat, soybeans

## Supplementary Materials

The following are available online at http://www.mdpi.com/2072-6643/10/4/464/s1, Table S1: Food grouping used in the dietary patterns analysis.
