# Supplementary material for: Dietary Patterns Associated with Cognitive Function among the Older People in Underdeveloped Regions: Finding from the NCDFaC Study
Source: Nutrients. 2018 Apr 9;10(4):464. doi: 10.3390/nu10040464 (PMC5946249; doi:10.3390/nu10040464)
Supplement: Supplementary file 1 [file nutrients-10-00464-s001.docx]

**Dietary Patterns Associated with Cognitive Function Among the Older People in Underdeveloped Regions: Finding from the NCDFaC Study**

**Supplementary file:**

**Table S1**. Food grouping used in the dietary pattern analysis.

| **Food Groups** | **Food Items/Subgroups** |
| --- | --- |
| cereal grains | rice product, wheat products, coarse cereals (millets, sorghum, maize, etc.) |
| tubers | tubers (sweet potatoes, Chinese yam, taro, potatoes, etc.) |
| fried foods | fried foods (deep-fried dough stick, fried dough cake, etc.) |
| red meat | pork, beef, and mutton |
| poultry | poultry |
| organ meat | organ meat |
| aquatic products | aquatic products |
| whole milk | whole milk |
| dairy products | milk powder, cheese, yogurt, |
| eggs | eggs |
| soybean products | tofu, bean curd/bean curd cake |
| soybean milk | soybean milk |
| dried legume | dried legume |
| vegetables | fresh vegetables, dried vegetables |
| pickles | salted vegetables, kraut, sauerkraut |
| fresh mushrooms | fresh mushrooms |
| dried mushrooms | dried mushrooms, other dried fungus (Chinese black fungus, tremella, bamboo fungus, etc.) |
| dessert | dessert |
| fresh fruits | fresh fruits |
| nuts | nuts |
| alcoholic beverages | low-alcohol liquor, strong liquor, beer, fruit wine |
| fruit and vegetables juice | fresh fruit and vegetable juice, packaged fruit and vegetables juice |
| beverages | sugar-contained beverages, less sugary drinks, coffee |
| tea | tea |
